# Spontaneous Renal Artery Dissection in a Normotensive Young Male

**DOI:** 10.1100/tsw.2004.87

**Published:** 2004-12-10

**Authors:** John A. Taylor, Eric C. Martin, Ihor S. Sawczuk

**Affiliations:** Columbia Presbyterian Hospital, USA

## Abstract

We report a case of spontaneous superior pole renal artery dissection in a healthy forty-one year old man. Spontaneous renal artery dissection is a rare event occurring in patients with hypertension or renovascular disease such as fibromuscular dysplasia. This case demonstrates the importance of maintaining renovascular accidents as part of the differential diagnosis of abdominal pain.

## CASE REPORT

A forty-one year old man presented to the emergency room complaining of sudden onset of abdominal pain followed by two episodes of emesis. The patient described periumbilical pain which intensified and migrated toward the right flank during the course of the day. The patient denied fever, dysuria, hematuria and gave no previous history of hypertension, arrhythmias, renal disease, nephrolithiasis or trauma. Laboratory data showed: white blood cell count (WBC)- 8.4, blood urea nitrogen/creatinine - 9/1.1, creatine phosphokinase (CPK)- 648, lactate dehydrogenase - 232, urinalysis with specific gravity of 1.005, pH 7.0, no albumin or protein, negative for ketones as well as leukocyte esterase and nitrites, and 6-10 red blood cells per high powered field. Furthermore, an electrocardiogram revealed normal sinus rhythm. All other values were within normal limits.

The elevated CPK had been attributed to intramuscular injections of pain medication. He was afebrile and normotensive. Initial examination revealed a positive Murphy's sign. Abdominal ultrasonography was performed and showed a normal gallbladder, no hydronephrosis and a possibly inflamed appendix. Subsequently, an abdomino-pelvic CT scan was performed which revealed hypoperfusion in the right superior renal pole with a patchy nephrogram (Figure 1).

A spontaneous dissection or embolus was suspected and the patient underwent a renal arteriogram. This demonstrated two renal arteries on the right with the superior pole vessel having an irregular appearance and a filling defect consistent with an intimal flap and thrombus (Figure 2).

Due to a warm ischemia time of greater than twenty-four hours from the onset of symptoms intervention was not pursued. A renal scan at this time demonstrated almost complete absence of activity in the right kidney. The following day the patient developed a post infarction syndrome with fever to 103 and a WBC of 18.0. Six months later, a renal scan showed 18% function on the right and 82% on the left.

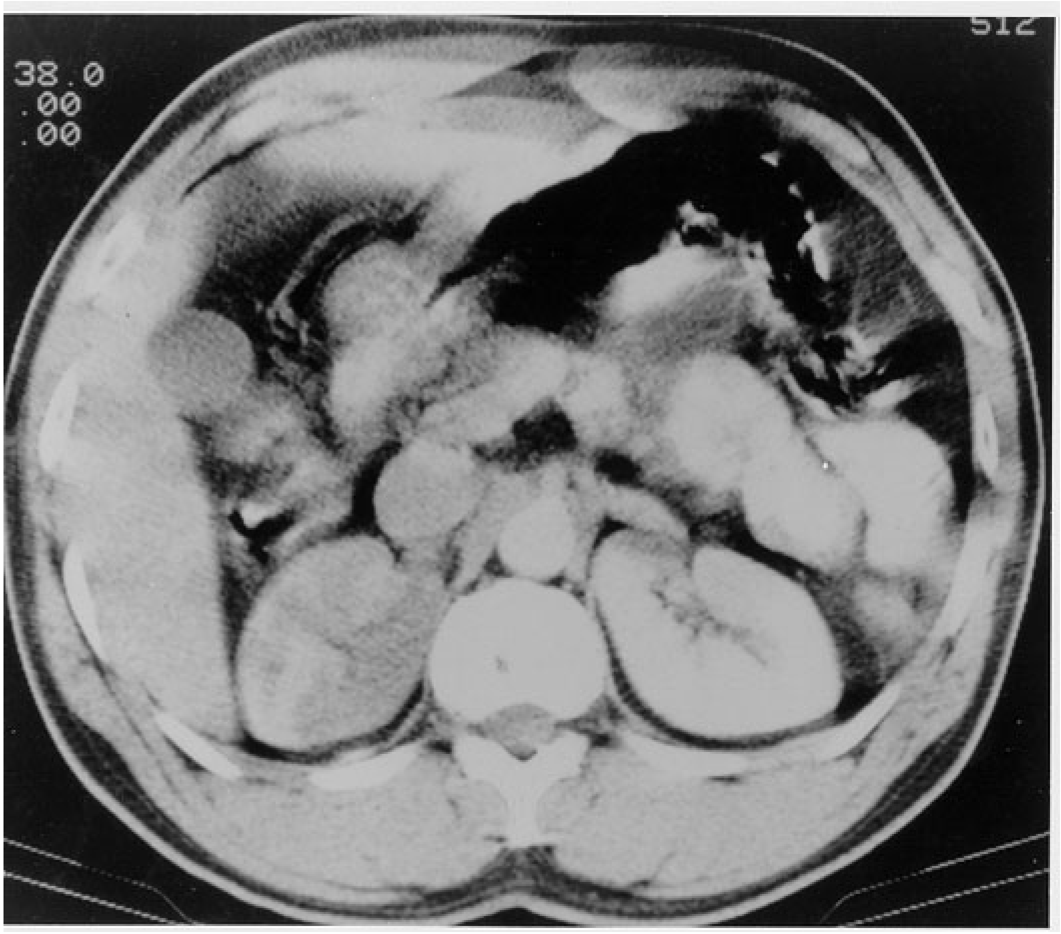

**FIGURE 1.** CT Scan of abdomen with intravenous and oral contrast.

Since this time the patient has been lost to follow-up and at last visit remained normotensive. His damaged kidney and vasculature remain in place thus pathologic evaluation is not available.

## DISCUSSION

Spontaneous renal artery dissection is a rare event, the natural history of which is unknown. The most consistent clinical sequence is the abrupt onset of severe, poorly controlled, persistent hypertension (HTN) and/or flank pain in a previously normotensive young man. Early recognition is imperative with keys to diagnosis including the above as well as the abrupt onset of proteinuria, abdominal bruits and/or renal failure (6). Although studies such as CT scan may permit diagnosis, high resolution arteriography is the study of choice to define clearly the anatomic lesion. Approximately ninety reported cases in the literature were reviewed, and none occurred in a patient without HTN. Dissection tends to occur in the absence of trauma and is typically associated with fibromuscular dysplasia (1) (FMD), atherosclerotic disease (3), aneurysmal disease (4) or HTN associated with renal pathology (5). The clinical course is notable for progression of HTN at times refractory to multiple medical regimens. There are only eight documented cases of reversion to normotension over intervals ranging from 2-45 months (5). These were associated with normalization of renin levels and/or arteriographic improvement.

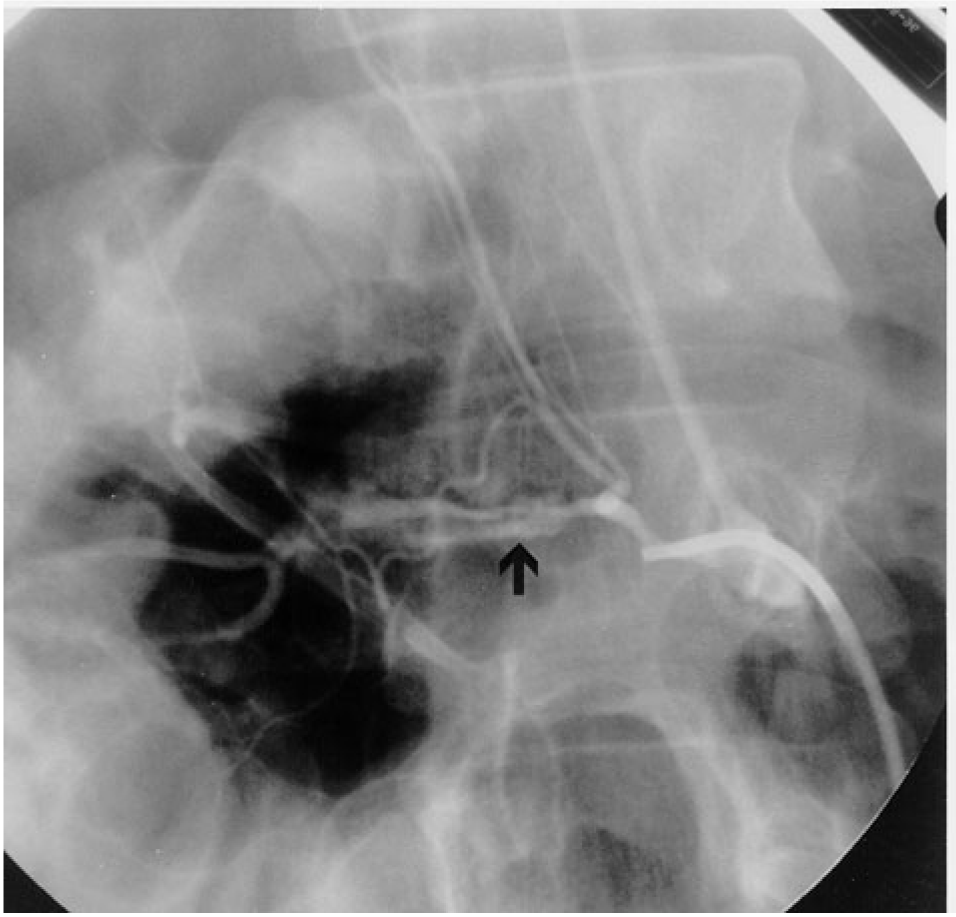

**FIGURE 2.** Right renal arteriogram. Arrow denotes area of dissection with thrombus.

Treatment modalities are limited and only efficacious if the process is recognized prior to irreversible parenchymal damage as dictated by the warm ischemia time. Some authors have reported thrombolytic therapy for post dissection thrombus however the problem of an anatomical defect, the intimal flap, still remains (6). The most common operative approach in over half of the reported cases was a partial or total nephrectomy. The concern with this approach is that FMD is bilateral in 30-50% of patients and that dissection has a bilateral component 20-25% of the time (5). The literature reports 32 vascular reconstructions attempted in 28 patients (5). Sixteen of the twenty-eight patients were considered a clinical success with 13 returning to previous pre-dissection blood pressure levels and three with improved control. Clinical and/or anatomical failures were noted in 9/28 patients and 3 were lost to follow-up(5). Reillyâs group also noted improvement in results with the use of autologous arterial grafts verses dacron or vein grafts. Five of seven cases had a return to previous dissection BP and prolonged renal function at a mean follow-up of 14.5 years.

The infrequency with which this disease entity is encountered makes large studies or reviews of treatment efficacy difficult. Vascular reconstruction with return to a relative normal anatomy with or without the use of thrombolytic therapy appears to be a viable consideration for the management of these patients. However, treatment can only be implemented in the early stages of renal ischemia. It is therefore important to keep this process in the differential when assessing abdominal pain of unclear etiology.
